# Integrating Spirituality Into Mental Health Care

**DOI:** 10.1192/bjo.2024.131

**Published:** 2024-08-01

**Authors:** Joanna Barber, Christopher Cook

**Affiliations:** 1 Birmingham and Solihull Mental Health NHS Foundation Trust, Birmingham, United Kingdom; 2 Durham University, Durham, United Kingdom

## Abstract

**Aims:**

To find how best to integrate religion/spirituality (R/S) into clinical care.

**Methods:**

This was a qualitative study. 41 mental health patients of varying diagnoses in secondary care underwent semi-structured interviews describing their mental health and spiritual journeys and how these have interacted, before, during and after a period of acute illness. Grounded theory was used. Detailed coding was carried out and themes extracted.

**Results:**

Preliminary results from this project have already been reported, (submitted for publication). 5 main processes by which R/S interacted positively or negatively with mental health recovery were identified:

- R/S experiences, (+ve or -ve),
- Existential crisis, (-ve),
- Influence of faith community, (+ve or -ve),
- Finding a personally meaningful faith, (+ve),
- Changing priorities to a more spiritual outlook, (+ve).

Further analysis has allowed a comparison between our different participants who were at different stages of recovery:

1. Those who described themselves most as being in recovery tended to have more positive R/S experiences, support from a faith community, a personally meaningful faith and have changed their priorities. Most have also found clinical care helpful. However, often R/S was considered more helpful both for personal recovery and symptom relief. For others in this group, R/S enables living a satisfying life despite limitations of illness partially controlled by medication.
2. Those who described themselves most as struggling with mental illness were much less likely to have a personally meaningful faith or had changed their priorities. They tended to have negative R/S experiences, persistent existential crisis and/or rejection from a faith community. Most of these people find both clinical care and R/S issues unhelpful. Some people were finding clinical care helpful but R/S barriers were blocking their recovery.

Many people at all stages of recovery said they wanted more help with R/S issues. They often regard their illness as a spiritual problem and consider positive R/S experiences a key to recovery.

**Conclusion:**

Spiritual health may be important for recovery from many mental health problems and needs to be addressed according to the 5 themes.

- Possible R/S barriers identified, even if symptoms seem to be responding to clinical treatment.
- Positive R/S experiences and/or support from a faith community used to help overcome R/S barriers.
- Support made available to find a personally meaningful faith and change priorities.
- Referral to spiritual care offered more frequently.

Clinical care will be most effective if combined with facilitating spiritual health.

